# Supplementary material for: Fun30 and Rtt109 Mediate Epigenetic Regulation of the DNA Damage Response Pathway in C. albicans
Source: J Fungi (Basel). 2022 May 25;8(6):559. doi: 10.3390/jof8060559 (PMC9225650; doi:10.3390/jof8060559)
Supplement: Supplementary file 1 [file jof-08-00559-s001.zip › jof-1663165-supplementary.pdf]

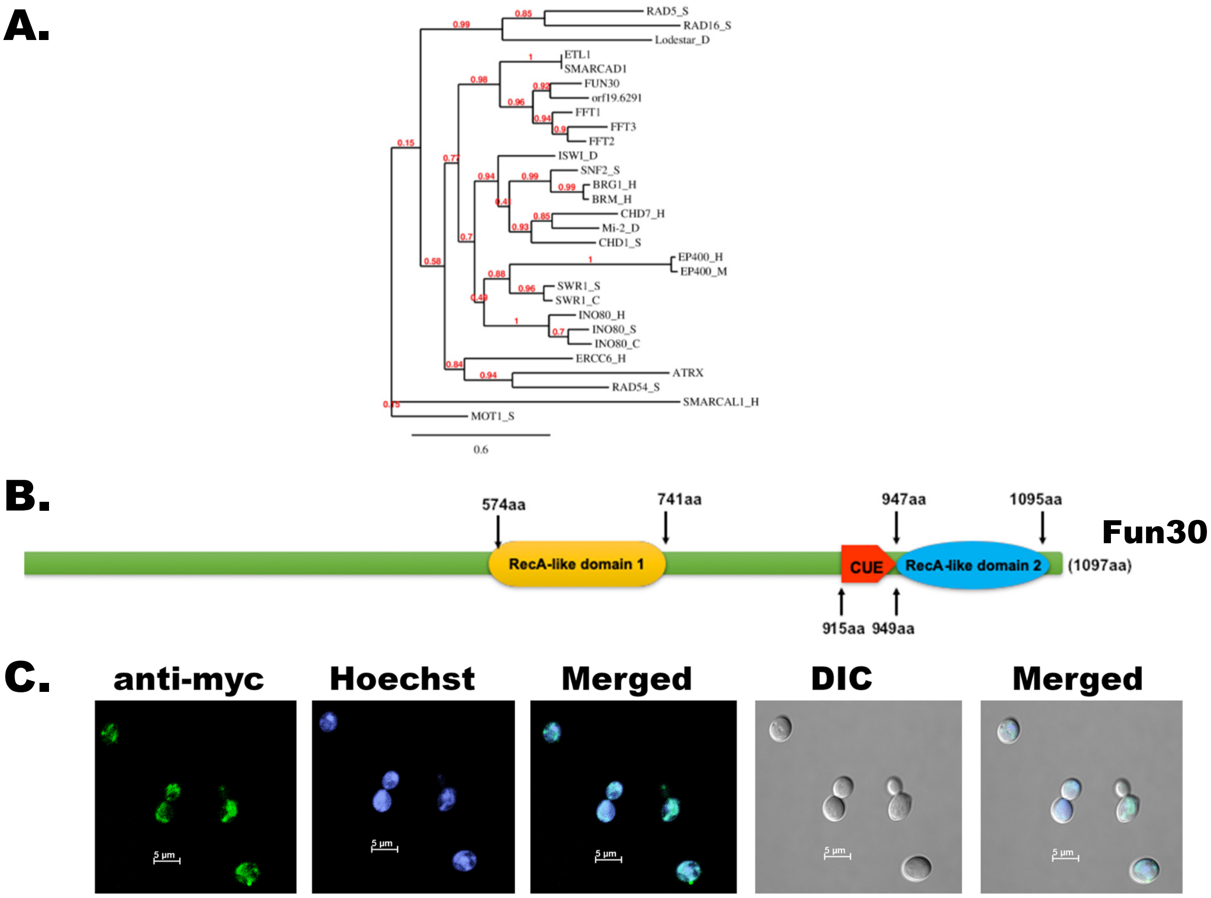

Figure S1. The orf19.6291 encodes for Fun30, a member of the ATP-dependent chromatin remodeling protein family. (**A**) Phylogenetic analysis of orf19.6291 protein from *C. albicans* was done with Rad5 from *S. cerevisiae* (RAD5_S), Rad16 from *S. cerevisiae* (RAD16_S), Lodestar from *D. melanogaster* (Lodestar D), Etl1 from *M. musculus* and SMARCAD1 from *H. sapiens*, Fun30 from *S. cerevisiae*, Fft1, Fft2, and Fft3 from *S. pombe* (FFT1, FFT2, and FFT3 respectively), Iswi from *D. melanogaster* (ISWI_D), Snf2 from *S. cerevisiae* (SNF2_S), BRG1, BRM, and CHD7 from *H. sapiens* (BRG1_H, BRM_H, CHD7_H respectively), Mi-2 from *D. melanogaster* (Mi-2_D), CHD1 from *S. cerevisiae* (CHD1_S), EP400 from *H. sapiens* and *M. musculus* (EP400_H and EP400_M respectively), SWR1 from *S. cerevisiae* and *C. elegans* (SWR1_S and SWR1_C respectively), Ino80 from *H. sapiens*, *S. cerevisiae* and *C. elegans* (INO80_H, INO80_S, and INO80_C respectively), ERCC6 from *H. sapiens* (ERCC6_H), ATRX from *H. sapiens*, Rad54 from *S. cerevisiae* (RAD54_S), SMARCAL1 from *H. sapiens* (SMARCAL1_H) and Mot1 from *S. cerevisiae* (MOT1_S). (**B**) Domain architecture of Fun30 (orf19.6291) from *C. albicans*. (**C**) Localization of Fun30 protein from *C. albicans* was monitored using immunofluorescence. Fun30 was tagged with myc and localization was monitored using anti-myc antibody. The nucleus was stained using Hoechst.

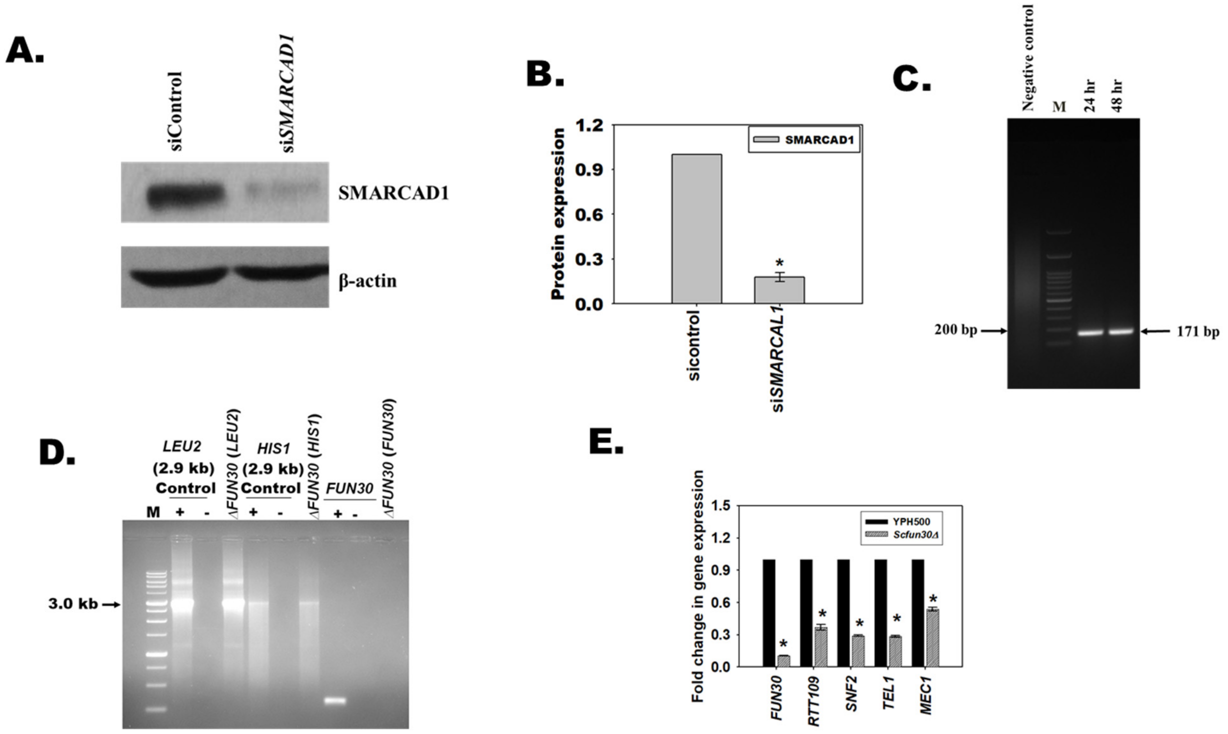

Figure S2. Confirmation of *FUN30* homozygous null mutant. **A.** Expression of SMARCAD1 was analyzed by western blot in si*SMARCAD1* cells. β-actin was used as the internal control. **B.** Quantification of the western blots**.** The data is represented as average ± s.d of two independent experiments. **C.** Expression of *C. albicans FUN30* was analyzed in si*SMARCAD1* cells at 24 hr and 48 hr. **D.** Confirmation of *ΔFUN30* mutant was done using PCR. **E.** Expression of *FUN30*, *RTT109*, *SNF2*, *TEL1*, and *MEC1* was analyzed by qPCR in YPH500 and *Scfun30Δ* mutants. Star indicates $p < 0.5$.

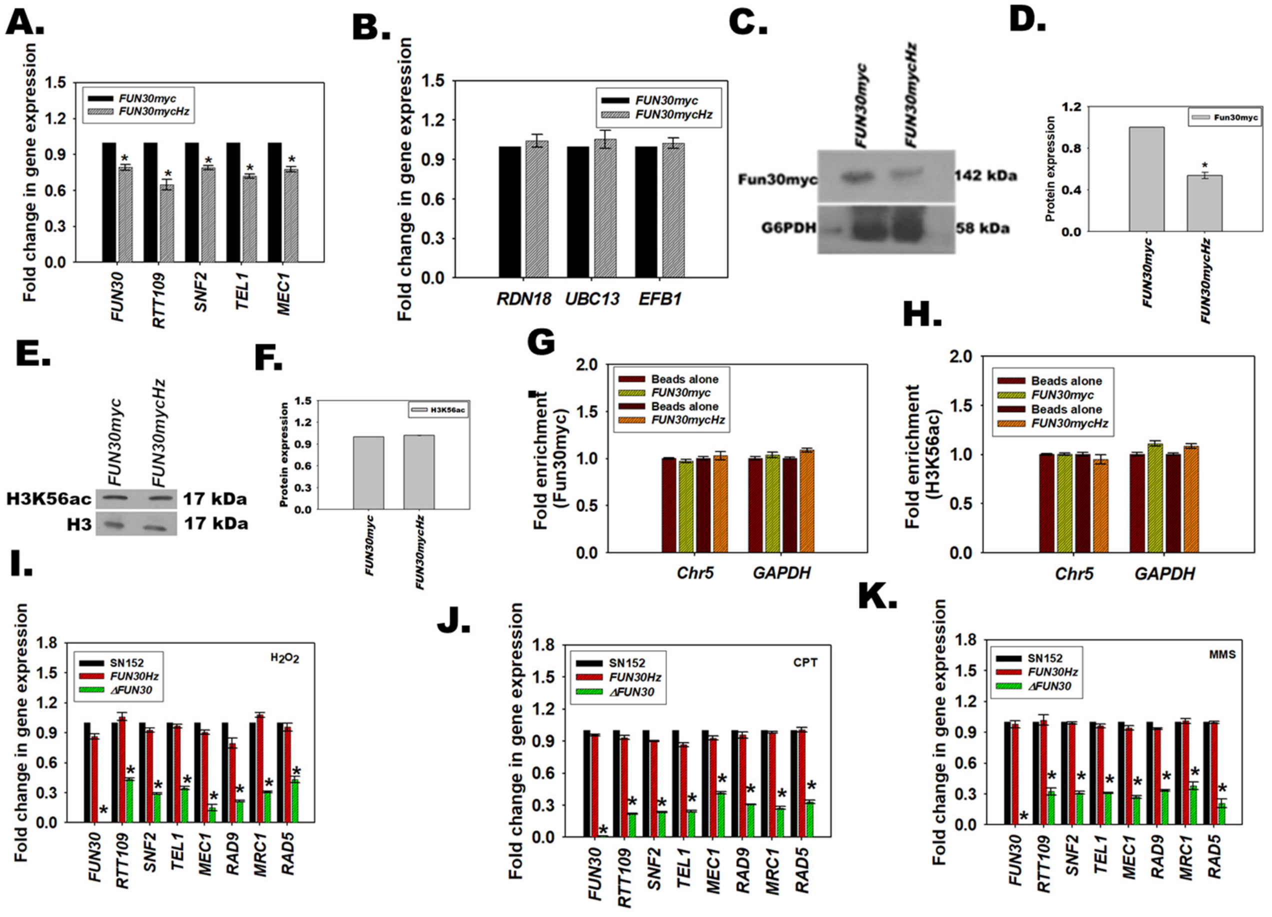

Figure S3. Expression of *RTT109, SNF2, TEL1,* and *MEC1* is regulated by Fun30. **A.** Expression of *FUN30, RTT109, SNF2, TEL1,* and *MEC1* was analyzed by qPCR in *FUN30myc* and *FUN30mycHz* mutants. **B.** Expression of *RDN18, UBC13,* and *EFB1* was analyzed by qPCR in *FUN30myc* and *FUN30mycHz* mutants. **C.** Western blots showing the expressing of Fun30 in *FUN30myc* and *FUN30mycHz* strains. G6PDH was used as loading control. **D.** Quantitation of Fun30myc expression in *FUN30myc* and *FUN30mycHz* strains from the western blots. **E.** Western blot showing the levels of H3K56ac in *FUN30myc* and *FUN30mycHz* strains. H3 was used as the loading control. **F.** Quantitation of H3K56ac expression in *FUN30myc* and *FUN30mycHz* strains from the western blots. **G.** Occupancy of Fun30myc on the intergenic region of Chr5 as well as *GAPDH* promoter in *FUN30myc* and *FUN30mycHz* strains. **H.** Occupancy of H3K56ac on the intergenic region of Chr5 as well as *GAPDH* promoter in *FUN30myc* and *FUN30mycHz* strains. **I.** Comparison of expression of *FUN30, RTT109, SNF2, TEL1, MEC1, RAD9, MRC1* and *RAD5* between SN152, *FUN30Hz,* and *ΔFUN30* in the presence of 7.5 μM H_2O_2. **J.** Comparison of expression of *FUN30, RTT109, SNF2, TEL1, MEC1, RAD9, MRC1,* and *RAD5* between SN152, *FUN30Hz,* and *ΔFUN30* in the presence of 1000 μM CPT. **K.** Comparison of expression of *FUN30, RTT109, SNF2, TEL1, MEC1, RAD9, MRC1,* and *RAD5* between SN152, *FUN30Hz,* and *ΔFUN30* in the presence of 0.02% MMS. Star indicates $p < 0.5$.

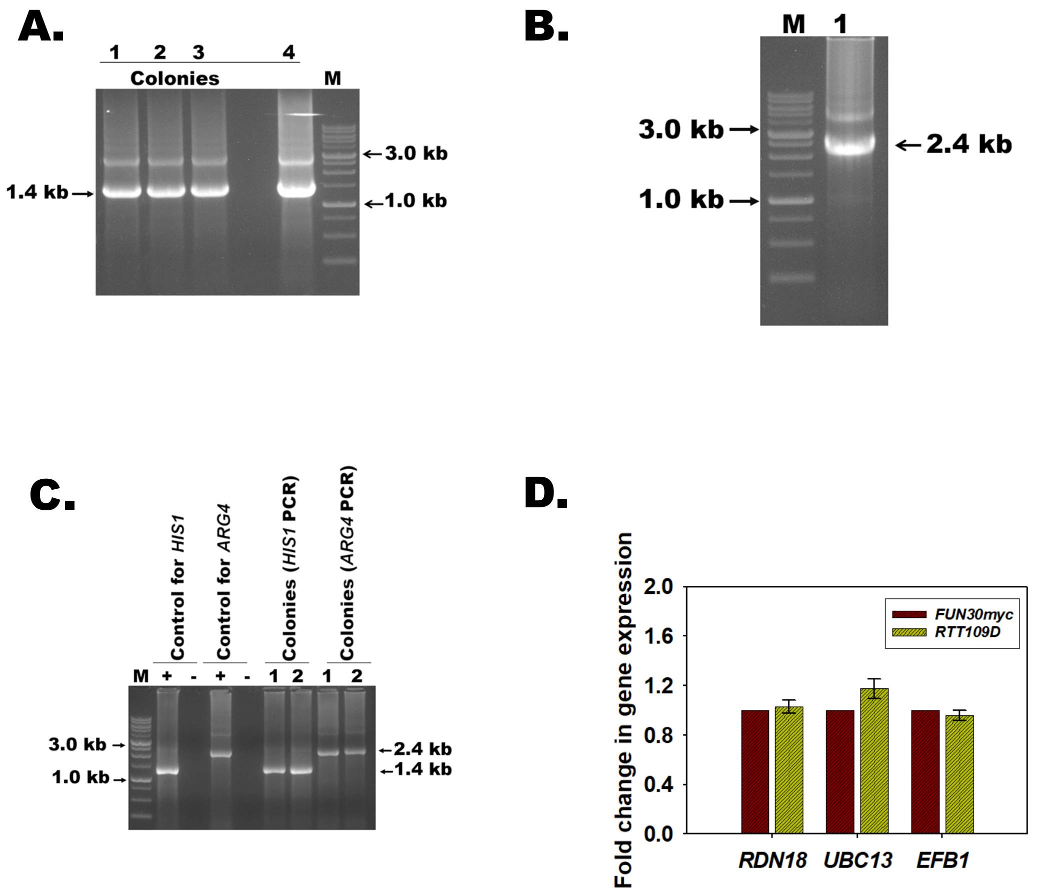

Figure S4. The *RTT109Hz* and *RTT109D* mutants were confirmed using PCR. **A.** One copy of *RTT109* was deleted in the BWP17 strain. Four colonies (# 1, 2, 3, and 4) were screened using *HIS1* specific primers and all the colonies gave the expected 1.4 kb product. **B.** The deletion of the second copy of *RTT109* in the *RTT109Hz* strain was confirmed by PCR using *ARG4* specific primers. The appearance of the expected 2.4 kb band confirmed the mutant strain. **C.** Deletion of the second copy of *RTT109* in *RTT109Hz* strain made in *FUN30myc* background was confirmed by PCR using *HIS1* and *ARG4* cassette specific primers. Two colonies (1 and 2) were screened. The appearance of the expected 1.4 kb and 2.4 kb confirmed the mutants. **D.** Expression of *RDN18*, *UBC13* and *EFB1* was analyzed by qPCR in *FUN30myc* and *RTT109D* strain.

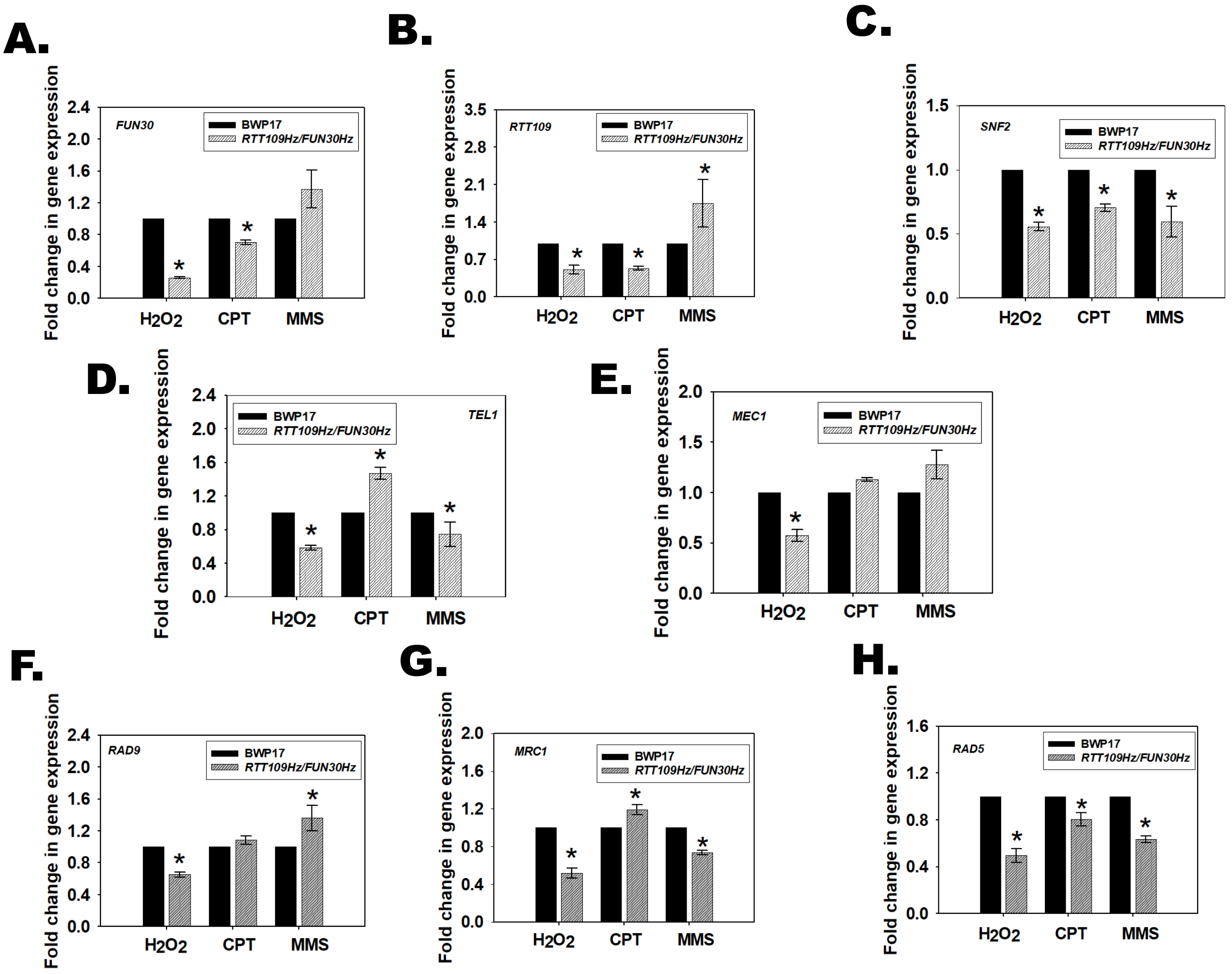

Figure S5. Rtt109 and Fun30 regulate the transcription of genes involved in DNA damage response pathways. Expression of **A.** *FUN30*; **B.** *RTT109;* **C.** *SNF2*; **D.** *TEL1*; **E.** *MEC1*; **F.** *RAD9*; **G.** *MRC1*; **H.** *RAD5* was analyzed in BWP17 and *RTT109Hz/FUN30Hz* strains in the presence of H_2O_2 (7.5 mM), CPT (100 µM), and MMS (0.02%). The qPCR data are presented as average ± s.e.m. of three biological replicates. Star indicates $p < 0.05$.

Genes	Untreated		H_2O_2 treated	CPT treated	MMS treated
	FUN30myc	*RTT109Hz/ FUN30mycHz*	*RTT109Hz/ FUN30mycHz*	*RTT109Hz/ FUN30mycHz*	*RTT109Hz/ FUN30mycHz*
FUN30	1	0.37	0.63	1.06	1.93
RTT109	1	0.55	0.56	0.92	2.37
SNF2	1	0.55	0.55	0.97	1.6
TEL1	1	0.57	0.51	0.96	1.97
MEC1	1	0.33	0.58	1.17	1.77
RAD9	1	0.58	0.68	1.22	2.13
MRC1	1	0.44	0.59	0.85	0.81
RAD5	1	0.46	0.48	0.93	0.54

<0.4 <0.5 <0.56 <0.6 <0.7 ~1 >1.5 >1.7 >1.9 >2

Figure S6. Expression (fold changes) of *FUN30*, *RTT109*, *SNF2*, *TEL1*, *MEC1*, *RAD9*, *MRC1*, and *RAD5* in *FUN30myc* and *RTT109Hz/FUN30mycHz* strains in untreated and treated conditions with H_2O_2 (7.5 mM), CPT (100 µM), and MMS (0.02%).

Promoters	Untreated		H_2O_2 treated		CPT treated		MMS treated	
	FUN30myc	*RTT109Hz/ FUN30mycHz*	*FUN30myc*	*RTT109Hz/ FUN30mycHz*	*FUN30myc*	*RTT109Hz/ FUN30mycHz*	*FUN30myc*	*RTT109Hz/ FUN30mycHz*
FUN30	1.88	0.97	1.86	0.9	0.75	1.11	1.17	0.93
RTT109	1.55	0.96	1.64	0.85	0.65	1.21	1.15	1.01
SNF2	1.52	0.67	1.48	0.78	0.83	1.16	1.17	1.15
TEL1	1.57	0.88	1.36	0.88	1.28	1.02	1.46	1.03
MEC1	1.95	0.75	2.25	1.06	0.82	0.94	1.05	1.05
RAD9	1.63	0.86	1.16	0.9	1.06	1	0.81	2.57
MRC1	1.98	0.7	6.05	1.3	1.18	1.2	1.41	0.81
RAD5	2.06	0.77	1.51	0.68	1.13	1.08	1.59	0.6

Decreased binding
Basal level of binding
Increased binding

Figure S7. The occupancy (fold changes) of Fun30myc on *FUN30*, *RTT109*, *SNF2*, *TEL1*, *MEC1*, *RAD9*, *MRC1*, and *RAD5* promoters in *FUN30myc* and RTT109Hz/*FUN30mycHz* strains in untreated and treated conditions with H_2O_2 (7.5 mM), CPT (100 µM) and MMS (0.02%).

Promoters	Untreated		H_2O_2 treated		CPT treated		MMS treated	
	FUN30myc	*RTT109Hz/ FUN30mycHz*	*FUN30myc*	*RTT109Hz/ FUN30mycHz*	*FUN30myc*	*RTT109Hz/ FUN30myc*	*FUN30myc*	*RTT109Hz/ FUN30myc*
FUN30	153.1	102.45	17.11	18.67	6.69	10.42	40.09	53.03
RTT109	198.49	126.2	75.52	70.66	27.07	49.31	11.02	6.49
SNF2	133.98	58.63	22.4	32.55	10.62	15.6	25.21	14.1
TEL1	173.47	118.17	51.29	57.09	24.69	15.32	7.69	21.01
MEC1	12.92	7.33	101.31	145.04	3.92	6.14	1.44	2.66
RAD9	146.22	92	25.55	18.05	13.23	17.23	1.08	1.71
MRC1	213.1	132.46	290.12	253.48	23.55	23.31	17.11	4.79
RAD5	192.33	129.37	154.2	131.93	25.27	33.22	39.38	15.95

Decreased binding
Basal level of binding
Increased binding

Figure S8. The occupancy (fold changes) of H3K56ac on *FUN30*, *RTT109*, *SNF2*, *TEL1*, *MEC1*, *RAD9*, *MRC1*, and *RAD5* promoters in *FUN30myc* and *RTT109Hz/FUN30mycHz* strains in untreated and treated conditions with H_2O_2 (7.5 mM), CPT (100 μM) and MMS (0.02%).

Table S1. List of primers used for constructing mutants.

Primer Name	Primer Sequence (5'→ 3')
RTT109-HindIII-FP	GCGAAGCTTGATTCTTAAAATCGCGTCCTTTCA
RTT109-NheI-RP	GCGGCTAGCCTATTTTGATTTTCTATAATTACT
RTT109-HIS1-FP	ATGCTTCCTCCAGATATATTACAAAATGGTGAATTTGAAA CCATTTACTTCCAGACAAATCGGGGATCCTGGAGGATGAG
RTT109-HIS1 -RP	CTATTTTGATTTTCTATAATTACTTACAAAATTACTAACT TCAACACGATCACTAAAATCCGGAATATTTATGAGAAACT
RTT109-ARG4-FP	ATGCTTCCTCCAGATATATTACAAAATGGTGAATTTGAAA CCATTTACTTCCAGACAAATTGTGGAATTGTGAGCGGAAG
RTT109-ARG4-RP	CTATTTTGATTTTCTATAATTACTTACAAAATTACTAACTT CAACACGATCACTAAAATCTTTCCCAGTCACGACGTT
FUN30-PstI-FP	GCGCTGCAGATGAGTTGGTTTAGAAGAAATAAAC
FUN30-NheI-RP	GCGGCTAGCTCAACTATAAACTATTGACTCTAATG
FUN30-HIS1-FP	ATGAGTTGGTTTAGAAGAAATAAACCAACGGAGGAATCA TCTACAGCTGATCCAAACACACGGGGATCCTGGAGGATGAG
FUN30-HIS1-RP	TCAACTATAAACTATTGACTCTAATGTTGAAATCTTTTCG TCTAACTCTAATTTGTTTCTCGGAATATTTATGAGAGAAACT
FUN30-ARG4-FP	ATGAGTTGGTTTAGAAGAAATAAACCAACGGAGGAATCA TCTACAGCTGATCCAAACACATGTGGAATTGTGAGCGGAAG
FUN30-ARG4-RP	TCAACTATAAACTATTGACTCTAATGTTGAAATCTTTTCG TCTAACTCTAATTTGTTTCTTTTCCCAGTCACGACGTT
FUN30-MYC-FP	GAAACAAATTAGAGTTAGACGAAAAGATTTCAACATTAGAGTCAATAGTTTATAGTCG-GATCCCCGGGTTAATTAACGG
FUN30-MYC-RP	TTACCAAAACAAGCGGGAAAATCAGCTTCAAATGTGTACATTTCTTATTTT-GCGGCGGCCGCTCTAGAACTAGTGGATC
RAD9-URA3-FP	ATACAATTGCCAGAAACTCAATCTCAAAGCTTATTATATTATGATTCACACTA-GAAGGACCACCTTTGATTG
RAD9-URA3-RP	TTTGGTGCTATAATTGGGATTATTTCATCTGATTGTTTTTAGATTCTGATTT-GTACAATTCATCCATAC
RAD5-HindIII-FP	CCCAAGCTTATGAAAGTTATAAAGAAAAGG
RAD5-Nhe1 -RP	CTAGCTAGCCTATTCCTCAAAAAGGATTTG
RPS1-RP	AATAGAGAGAAACTATATTATACAC
FUN30 URA3 FP	TTGTCGTGAAAAATTGAGACACGACCTGCATTTCAGTCGACGCGTGTTTTTCAATTCTA-GAAGGACCACCTTTGATTGT

FUN30 MET3 RP	TTCCTCCGTTGGTTTATTTCTTCTAAACCAACTCATATTGAATGAAAATAAGTAAA-GCCATTTTAATAAACGCGGATCC
FUN30-Xho1-FP	CCGCTCGAGATGAGTTGGTTTAGAAGAAATAAAC
FUN30-Xma1-RP	CCCCCCGGGTCAACTATAAACTATTGACTCTAATG
FUN30-BamH1	CGCGGATCCATGAGTTGGTTTAGAAGAAATAAAC
FUN30-Mlu1-RP	CGACGCGTTCAACTATAAACTCCATTGACTCTAATG
ScFUN30 deletion-FP	ATGAGTGGTTCGCATTCAAATGATGAGGATGACGTAGTGCAAGTGCCCGAGAC-GTCCTCTCTCCAGGAACCGAAATACA
ScFUN30 deletion-RP	TTATTCTTTGGTTCCCTTCGGTTTCGAGTTTTCATCATAAATTATATCCTCCAACATA-TCCTCAACCAAGTCATTCTGAG
ScDownFun30-FP	GATGAAAACTCGAAACCGAAGGGAACCAAAGAATAAATAAATAAAAA-TATAGTAACTCCAGGAACCGAAATACA
ScDownFun30-RP	GCTATACATACTAACCTATGTATATATACACATATATGTATATTATACTCTA-GAACTCAACCAAGTCATTCTGAG
PHO5-FP	TACCATTCCCTTAGGCAAAC
PHO5-RP	CATGTCTACCAACCATTTGC
FUN30-GKT-FP	GCACGAGATGGGTCTTGGTGCAACTTGTCAAGTCATTGCA
FUN30-GKT-RP	TGCAATGACTTGACAAGTTGCACCAAGACCCATCTCGTCG
FUN30 Primer 1	ATCGCTAAGTATCGCCCGTT
FUN30 Primer 3	cacggcgcgcctagcagcggTGGATGGAGATGGCTTGGTT
FUN30 Primer 4	gtcagcggccgcatccctgcAGCTGATTTTCCCGCTTGTT
FUN30 Primer 6	TGTCAATCCACCACCACCAA
Universal Primer 2	ccgctgctaggcgcgccgtgACCAGTGTGATGGATATCTGC
Universal Primer 5	gcagggatgcggccgctgacAGCTCGGATCCACTAGTAACG

Table S2. List of plasmids used in this study.

Plasmids	Reference
p*ACT1-GFP*	Alistair Brown, Aberdeen
p*ACT1-RTT109*	This study
p*ACT1-FUN30*	This study
p*ACT1-RAD5*	This study
pmCherry-*HIS1*	Victoria et al. [1]
pRS-*ARG4*	Wilson et al. [2]
URA3-p*MET3-GFP*	Gerami-Nejad et al. [3]
Yep*HIS*	Jain et al. [4]
pYES2	This study
eGFP-C2	This study
pSN40	This study
pSN52	This study

Table S3. List of strains used in this study.

Strains	Genotype	Referred in Manuscript	Reference
**Mutants made using BWP17 strain**			
BWP17	*ura3*::imm434/*ura3*::imm434 *iro1*/*iro1*:: imm434 *his1*::hisG/*his1*::hisG*arg4*/*arg4*	BWP17	Wilson et al., 1999 [2]
BWP17-*URA3*	BWP17 with *RPS1*/*rps1Δ*:: *pACT1-GFP*::*URA3*	BWP17-*URA3*	Jain et al., 2010 [4]
VPS75/*vps17-URA3*	BWP17with *VPS75*/*vps75Δ*::*ARG4* with *RPS1*/*rps1Δ*::*pACT1-GFP*::*URA3*	*VPS75Hz-URA3*	This study
RTT109/*rtt109*	BWP17 with *RTT109*/*rtt109Δ*::*HIS1*	*RTT109Hz*	This study
RTT109/*rtt109-URA3*	BWP17-*RTT109* heterozygous with *RPS1*/*rps1Δ*::*pACT1-GFP*::*URA3*	*RTT109Hz-URA3*	This study
rtt109/*rtt109-URA3*	BWP17-*RTT109* heterozygous *URA3* with *RTT109*/*rtt109Δ*::*ARG4*	*RTT109D-URA3*	This study
rtt109/*rtt109*/ p*ACT1-RTT109*	BWP17-*RTT109* deletion with *RPS1*/*rps1Δ*::*pACT1-RTT109*	*RTT109D*/ *RTT109OE-URA3*	This study
rtt109/*rtt109*/ p*ACT1-FUN30*	BWP17-*RTT109* deletion with *RPS1*/*rps1Δ*::*pACT1-FUN30*	*RTT109D*/ *FUN30OE-URA3*	This study
FUN30/*fun30*	BWP17 with *FUN30*/*fun30Δ*::*HIS1*	*FUN30Hz*	This study
FUN30/*fun30-URA3*	BWP17- *FUN30* heterozygous with *RPS1*/*rps1Δ*::*pACT1-GFP*::*URA3*	*FUN30Hz-URA3*	This study
FUN30/*fun30*/ p*ACT1-FUN30*	BWP17-*FUN30* heterozygous with *RPS1*/*rps1Δ*::*pACT1-FUN30*	*FUN30Hz*/ *FUN30OE-URA3*	This study
FUN30/*fun30*/ p*ACT1-RTT109*	BWP17-*FUN30* heterozygous with *RPS1*/*rps1Δ*::*pACT1-RTT09*	*FUN30Hz*/ *RTT109OE-URA3*	This study
RTT109/*rtt109*/ *FUN30*/*fun30*	BWP17-*RTT109* heterozygous with *FUN30*/*fun30Δ*::*ARG4*	*RTT109Hz*/ *FUN30Hz*	This study
RTT109/*rtt109*/ *FUN30*/*fun30 -URA3*	BWP17-*RTT109* and *FUN30* heterozygous with *RPS1*/*rps1Δ*::*pACT1-GFP*::*URA3*	*RTT109Hz*/ *FUN30Hz -URA3*	This study
RTT109/*rtt109*/ *FUN30*/*fun30*/ *RAD9*/*rad9-URA3*	BWP17-*RTT109* and *FUN30* heterozygous with *RAD9*/*rad9Δ*::*URA3*	*RTT109Hz*/ *FUN30Hz*/ *RAD9Hz-URA3*	This study
RTT109/*rtt109*/ *FUN30*/*fun30*/ p*ACT1-RAD5*	BWP17-*RTT109* and *FUN30* heterozygous with *RPS1*/*rps1Δ*::*pACT1-RAD5*	*RTT109Hz*/ *FUN30Hz*/ *RAD5OE-URA3*	This study
**Mutants made using SN152 strain**			
SN152	*ura3*/::imm434::*URA3*/*ura3*::imm434 *iro1*::*IRO1*/*iro1*::imm434 *his1*::hisG/*his1*::hisG *leu2*/*leu2 arg4*/*arg4*	SN152	Noble and Johnson, 2005 [5]
SN152/*fun30*	SN152 with *FUN30*/*fun30Δ*::*LEU2*	*FUN30Hz*	This study
fun30/*fun30*	SN152 with *fun30Δ*::*LEU2* /*fun30Δ*::*HIS1*	*ΔFUN30*	This study
FUN30myc	SN152 with *FUN30*-13X myc-*FLP-SAT1*	*FUN30myc*	This study
RTT109/*rtt109*	*FUN30myc* with *RTT109*/*rtt109Δ*::*HIS1*	*RTT109Hz*	This study
FUN30myc/*fun30*	*FUN30myc* with *FUN30*/*fun30Δ*::*HIS1*	*FUN30mycHz*	This study
rtt109/*rtt109*	*FUN30myc-RTT109* heterozygous with *RTT109*/*rtt109Δ*::*ARG4*	*RTT109D*	This study
RTT109/*rtt109*/ *FUN30myc*/*fun30*	*FUN30myc-RTT109* heterozygous with *FUN30*/*fun30Δ*::*ARG4*	*RTT109Hz*/ *FUN30mycHz*	This study
**Mutants made in *S. cerevisiae***			
YPH500	MAT*α* ura3-52 lys2-801_amber ade2-101_ochre trp1-Δ63 his1-Δ200 leu2-Δ1	YPH500	Sikorski and Hieter, 1989 [6]
JKM179	MAT*α* ade1 leu2-3,112 lys5 trp1::hisG ura3-52 hml::ADE1 hmr::ADE1 ade3::GAL::HO	JKM179	Eapen et al., 2012 [7]
Scfun30Δ	YPH500 with *fun30Δ*::*URA3*	*fun30Δ*	This study

fun30Δ	JKM179 with *fun30Δ*::*URA3*	*fun30Δ*	This study
YPH500-*URA3*	YPH500; intergenic region::*URA3*	YPH500-*URA3*	This study
JKM179-*URA3*	JKM179; intergenic region::*URA3*	JKM179-*URA3*	This study
YPH500-*URA3*-Yep*HIS*	YPH500; intergenic region::*URA3* with Yep*HIS*	YPH500-*URA3*-YepHIS	This study
JKM179-*URA3*-Yep*HIS*	JKM179; intergenic region::*URA3* with YepHIS	JKM179-*URA3*-YepHIS	This study
Scfun30Δ-Yep*HIS*	YPH500; *fun30Δ*::*URA3* with Yep*HIS*	*fun30Δ*-Yep*HIS*	This study
fun30Δ-Yep*HIS*	JKM179; *fun30Δ*::*URA3* with Yep*HIS*	*fun30Δ*-Yep*HIS*	This study
Scfun30Δ-Yep*HIS-Ca-FUN30*	YPH500; *fun30Δ*::*URA3* with Yep*HIS*-Ca*FUN30*	*fun30Δ*-Yep*HIS*-Ca-*FUN30*	This study
fun30Δ-Yep*HIS*-Ca-*FUN30*	JKM179; *fun30Δ*::*URA3* with Yep*HIS*-Ca*FUN30*	*fun30Δ*-Yep*HIS*-Ca-*FUN30*	This study

Table S4. List of primers used for qPCR analysis.

	Forward Primer (5'→ 3')	Reverse Primer (5'→ 3')
	C. albicans	
RTT109	TCGTTGATTGGATGCTGTAAGG	ACCAGCTTCAACAGGTTCATAA
FUN30	GTGGAATTGAACCAAGTGTAGCTG	TGAGATTGCCTTCCGTTGTCTC
TEL1	ATTCTACCAGTTGGCTTGCGA	TCCAAAGTTGTTCTTTCCGGC
MEC1	GAGACACAGCAAGACCCATTA	CGAGCAACTTGTCATCTTTCAG
SNF2	TGGAGGAGTATGGTCGTGGT	TCTTCGGCTTGGCTTCCATT
RAD5	GGCCATACGCATCTCAAACT	CGGCGTTTGGATAATCTTGT
RAD9	TCAAATTCATTGGTGGCTGA	TTCGTTTTCGTTCTCGTTCA
MRC1	TGACGAACAAGCCACTCAAG	TCATTTTACGACCACGACGA
UBC13	TCGGTCCTAATCAATCACCTT	TCTTTCAACACATCCAAACAAA
RDN18	CCACCACCCACAAAATCAA	CGGCACCTTACGAGAAATCA
EFB1	GCTTCTGGTTCTGCTGCTG	GCTGGTTTTGGACCTTTAGC
VPS75	CCAATGTATGCCAAAAGACG	AATGACCTGCCTCATCATCG
GAPDH	GTCCATCCCACAAGGACTGG	CAACGGAAACATCGGTGGTTGG
	S. cerevisiae	
RTT109	GTACGCCAGCAAGAACTTTA	AGAATCTGGGAAGAGGTACTG
FUN30	GCCTCACATAGGCCATATTC	CAGACTCAATTGCATTGTAACC
TEL1	GTTCGATACGAGGCCTTTAG	TCAGGTCCGATTTCTCTTTG
MEC1	GTTCGATACGAGGCCTTTAG	TCAGGTCCGATTTCTCTTTG
SNF2	ACGACGACGACAATTCTAAC	CTTAAGGTAGGAGCCCATTTC
ALG9	GTGGCTTTGGTGAACAATTAC	CCTGGAAGAAGACCATCAAAT
	**Chr3 Primers used for Double-strand end resection**	
Distance (kb)		
−56.59	CCAAAGCGTCATGGACATCT	AGGCCCATCATTCTACTACTGG
−48.148	CACGCCTAGTTTCAGCTTGTTT	CTTCAAGACATAATCAACGACGC
−29.91	TCGTCGTCGCCATCATTTTC	GCCCAAGTTTGAGAGAGGTTGC
−16.712	CGTCTTCTCAGCGAACAACAGC	CGTCTTCTCAGCGAACAACAGC
−9.592	TCAGGGTCTGGTGGAAGGAATG	CAAAGGTGGCAGTTGTTGAACC
−5.358	ATTGCGACAAGGCTTCACCC	CACATCACAGGTTTATTGGTTCCC
−0.186	CATACAGAAACACAGCGG	AGGAAGGAACAGGAATCTGG
0.183	CCTGGTTTTGGTTTTGTAGAGTGG	GAGCAAGACGATGGGGAGTTTC
5.453	GGACTGGTTATAGACGATGAAGTGT	AAGTCGTCCTTCTTCTTGCTCC
9.187	TGGATCATGGACAAGGTCCTAC	GGCGAAAACAATGGCACTCT
18.206	CGGTCCTCGATTTTGTTACCTTC	GCAAGGATATTCCTGCCTTTTTC
26.869	GGAAAGACTGGCTCATCAAAAC	ACATTCTCAGAGAGAACCTCCA
47.693	ACACCCTGAATGGGGAAAC	CTGCATGGGTGCTTGATG
61.721	ATCTCAGCCAGCTGCTGG	CCCTCTATCTGTCCTTCTGC
69.192	AACAACGGTGAACGGTGCTG	GCAGTAGAAACCTGGGATGTGG

Table S5. List of primers used for ChIP analysis.

Gene Promoter	Forward Primer (5'→ 3')	Reverse Primer (5'→ 3')
FUN30	AATCAGTTGTATAGGAAGGAGAT	GCAAAAGTTGGTTATTTGTACTAG
SNF2	GATGAGGGTGGTTGGAGATAT	GGATGAATACTATGTATTGGGTCG
MEC1	ATTATTCAAGAGACCCTTAAATCCC	ATTTCTATCCTATTTCTGAGGAGGG
TEL1	GGTAGAGAGAGTGACAGTATCAACT	ATATCTGACGTAGACATGATTACG
RTT109	CCATCCCAGTTAATTGTTTACCATCTG	GAATACACCACAATAACAACACTTGCTAAT
RAD5	CCAACCTTCACTCTAACAAACATTTAG	TGGGAGAAGTGGTTGTTTCTTC
RAD9	AGTGTGCACAACTTGAATGG	GTGAATTCTTTGATGAGGCAGAT
MRC1	GCAGATCCCAATTTGTAACACT	CGTACGGTTTAGCTTAGAAAGAAA
Chr5 inter-genic region	ATGGGTGTGCTGCTTTTGTT	TAAGGAAGTTGTCGGGTCGA
GAPDH	CAGCTGTTTCAAATCCAGGCT	GTGGTTGAGTGGGTTGGTTG

Table S6. Doubling time for wild-type and mutant strains in the absence and presence of MMS.

Strain	Doubling Time (hr)	*P*-Value (Significance with Respect to BWP17-*URA3*)
**Without MMS**		
BWP17-URA3	1.8 ± 0.04	-
RTT109Hz/FUN30Hz-URA3	2.4 ± 0.004	5.68E-03
RTT2109Hz/FUN30Hz/RAD5OE-URA3	2.5 ± 0.02	5.04E-03
RTT109Hz/FUN30Hz/RAD9Hz-URA3	1.8 ± 0.02	0.06 (not significant)
**With MMS**		
BWP17-URA3	3.9 ± 0.04	-
RTT109Hz/FUN30Hz-URA3	2.4 ± 0.09	4.68E-03
RTT2109Hz/FUN30Hz/RAD5OE-URA3	3.2 ± 0.07	5.14E-03
RTT109Hz/FUN30Hz/RAD9Hz-URA3	3.8 ± 0.02	0.31 (not significant)

References

1. Victoria, G.S.; Kumar, P.; Komath, S.S. The Candida Albicans Homologue of PIG-P, CaGpi19p: Gene Dosage and Role in Growth and Filamentation. *Microbiology (Reading)* **2010**, *156*, 3041–3051, doi:10.1099/mic.0.039628-0.
2. Wilson, R.B.; Davis, D.; Mitchell, A.P. Rapid Hypothesis Testing with Candida Albicans through Gene Disruption with Short Homology Regions. *Journal of Bacteriology* **1999**.
3. Gerami-Nejad, M.; Hausauer, D.; McClellan, M.; Berman, J.; Gale, C. Cassettes for the PCR-Mediated Construction of Regulatable Alleles in Candida Albicans. *Yeast* **2004**, *21*, 429–436, doi:10.1002/yea.1080.
4. Jain, P.; Garai, P.; Sethi, S.C.; Naqvi, N.; Yadav, B.; Kumar, P.; Singh, S.L.; Yadav, U.; Bhatnagar, S.; Rahul; et al. Modulation of Azole Sensitivity and Filamentation by GPI15, Encoding a Subunit of the First GPI Biosynthetic Enzyme, in Candida Albicans. *Scientific Reports* **2019**, doi:10.1038/s41598-019-44919-4.
5. Noble, S.M.; Johnson, A.D. Strains and Strategies for Large-Scale Gene Deletion Studies of the Diploid Human Fungal Pathogen Candida Albicans. *Eukaryotic Cell* **2005**, doi:10.1128/EC.4.2.298-309.2005.
6. Sikorski, R.S.; Hieter, P. A System of Shuttle Vectors and Yeast Host Strains Designed for Efficient Manipulation of DNA in Saccharomyces Cerevisiae. *Genetics* **1989**, *122*, 19–27.
7. Eapen, V.V.; Sugawara, N.; Tsabar, M.; Wu, W.-H.; Haber, J.E. The Saccharomyces Cerevisiae Chromatin Remodeler Fun30 Regulates DNA End Resection and Checkpoint Deactivation. *Mol. Cell. Biol.* **2012**, *32*, 4727–4740, doi:10.1128/MCB.00566-12.
